# Supplementary material for: A multi-view pipeline and benchmark dataset for 3D hand pose estimation in surgery
Source: Int J Comput Assist Radiol Surg. 2026 May 11;21(6):1217–25. doi: 10.1007/s11548-026-03610-6 (PMC13690852; doi:10.1007/s11548-026-03610-6)
Supplement: Supplementary file 1 [file 11548_2026_3610_MOESM1_ESM.pdf]

A Multi-View Pipeline and Benchmark Dataset for 3D Hand Pose Estimation in Surgical Environments

International Journal of Computer Assisted Radiology and Surgery

Online Resource 1

Valery Fischer[1,2*], Alan Magdaleno[1,2], Anna-Katharina Calek[1], Nicola Cavalcanti[1], Nathan Hoffman[1], Christoph Germann[1], Joschua Wüthrich[1], Max Krähenmann[1], Mazda Farshad[1], Philipp Fürnstahl[1], Lilian Calvet[1]

[1]University Hospital Balgrist, University of Zurich, Switzerland.
[2]ETH Zürich, Switzerland.

*Corresponding author(s). E-mail(s): vfischer@ethz.ch;

This online resource 1 provides additional technical details, extended experimental results, and comprehensive information about the proposed dataset that could not be included in the main manuscript due to space constraints. Section 1 defines the evaluation metrics used throughout our experiments. Section 2 details the dataset and acquisition setup. Additional qualitative and quantitative results are reported in Section 3. Finally, Section 4 details implementation aspects and parameter settings for reproducibility.

1 Evaluation scores

To comprehensively assess the performance of our proposed method, we employ a set of standard quantitative metrics commonly used in hand and human pose estimation tasks.

Mean Per Joint Position Error (MPJPE) This metric measures the mean Euclidean distance between predicted and ground-truth 3D joint positions:

$$\text{MPJPE} = \frac{1}{NJ} \sum_{i,j,m=1}^{N,J} \left\| \hat{\mathbf{X}}_{i,j} - \mathbf{X}_{i,j} \right\|_2 , \tag{1}$$

where N is the number of frames, J is the number of joints, $\hat{\mathbf{X}}_{i,j}$ is the predicted 3D position of joint j in frame i, and $\mathbf{X}_{i,j}$ is the corresponding ground truth.

Mean Reprojection Error (MRE) This metric evaluates how well the predicted 3D joints project back into the 2D image space:

$$\mathrm{MRE} = \frac{1}{NVJ} \sum_{i,j,v=1}^{N,J,V} \left\| \Pi_v(\hat{\mathbf{X}}_{i,j}) - \mathbf{x}_{i,j,v} \right\|_2, \tag{2}$$

where $\Pi_v(\cdot)$ is the projection function using the intrinsic and extrinsic parameters of view v, and $\mathbf{x}_{i,j,v}$ is the ground-truth 2D position in view v.

It is important to note that, since both MPJPE and MRE are mean distance-based evaluation scores, they are particularly sensitive to outliers, as even a small number of poorly localized joints can significantly increase the overall error.

Percentage of Correct Keypoints in 2D (PCK_{2D}) $PCK_{2D}@\tau$ measures the fraction of 2D keypoints with reprojection error below a pixel threshold τ:

$$PCK_{2D}@\tau = \frac{1}{NVJ} \sum_{i,j,v=1}^{N,J,V} 1\left[\left\| \Pi_v(\hat{\mathbf{X}}_{i,j}) - \mathbf{x}_{i,j,v} \right\|_2 < \tau \right] \tag{3}$$

where $1[\cdot]$ is the indicator function.

Percentage of Correct Keypoints in 3D (PCK_{3D}) $PCK_{3D}@\delta$ evaluates the percentage of 3D joints with Euclidean error less than a threshold δ (in mm):

$$PCK_{3D}@\delta = \frac{1}{NJ} \sum_{i,j=1}^{N,J} 1\left[\left\| \hat{\mathbf{X}}_{i,j} - \mathbf{X}_{i,j} \right\|_2 < \delta \right]. \tag{4}$$

Mean Percentage of Correct Keypoints Additionally we use the expression mean PCK ($mPCK$) to denote the Mean Percentage of Correct Keypoints in either 2D or 3D. For 2D the threshold values are 5, 10, 20 and 30, and for 3D the values correspond to 5, 10, 25, 50, from which the mean is taken, similar to the evaluation protocol [1]. The unit is either mm for $mPCK_{3D}$ and pixel for $mPCK_{2D}$.

Average Precision (AP) To evaluate detection quality in 2D pose estimation, we also compute AP score across multiple keypoint thresholds, following the COCO evaluation protocol [1]:

$$\mathrm{AP} = \frac{1}{K} \sum_{k=1}^{K} \mathrm{Precision}(k) \tag{5}$$

where K is the number of thresholds (from 0.5 to 0.95 in 0.05 increments), and $\mathrm{Precision}(k)$ is computed at each threshold using matching between predicted and ground truth keypoints based on Object Keypoint Similarity (OKS) reported in [1].

Precision(k) computes the ratio of keypoints within the OKS threshold to the total number of keypoints that were predicted by the estimator. This formulation highlights that AP is computed only over predicted keypoints and therefore does not explicitly penalize missed detections, in contrast to PCK scores, which is central to the interpretation of our results in Table 1 of the main paper.

Mean Joint Error (MJE) We report mean joint error for the evaluation of 2D poses in the image space, giving the mean error for the hand poses given ground truth poses:

$$\text{MJE} = \frac{1}{NVJ} \sum_{i,j,v=1}^{N,J,V} \left\| \hat{\mathbf{x}}_{i,j,v} - \mathbf{x}_{i,j,v} \right\|_2 . \tag{6}$$

These metrics jointly provide a robust evaluation of the geometric accuracy of the estimated hand poses, both in image and 3D space for the full pipeline, as well as the detectability and localization precision of individual keypoints.

2 Dataset

This section details the experimental datasets used to evaluate the proposed methods, encompassing both controlled non-invasive and anatomically realistic ex-vivo surgical scenarios.

SPP (Spinal Preparation; non-invasive mock patient) A resident orthopedic surgeon simulated the pre-incision phase of a midline lumbar approach on a volunteer mock patient. The sterile field was prepared with standardized surgical drapes (IVF Hartmann, Neuhausen am Rheinfall, Switzerland) and Ioban incise foil (3M, Maplewood, MN, USA). No cuts, resections, or instrumentation were performed. This scenario isolates lighting, draping, and staff-induced occlusions without tissue deformation from dissection, providing a controlled yet realistic background for pose-estimation benchmarking.

SPI (Spinal Instrumentation; ex-vivo) A cadaveric midline lumbar approach (fresh-frozen torso, Th1–coccyx; male, 76 years, BMI 21.7 kg/m^2) was performed by an attending surgeon with a resident orthopedic surgeon. The specimen (Science Care, Arizona, US) was stored at $-22°$C and thawed for 72 h at room temperature at OR-X, Zurich, prior to use. The torso was placed on a commercial height-adjustable OR table (Hillrom, PST 500), draped as in SPP, and hydrated with periodic moist gauze coverage. The approach exposed L1–L5 with selective soft-tissue removal to access dorsal bony landmarks (mammillary processes, facet joints, laminae, spinous processes). Pedicle access followed routine practice using an awl and luer; Pennybacker and Cobb instruments were used as needed. Canal integrity was verified with a pedicle feeler before temporary placement of standardized polyaxial head cannulated pedicle screws (6×50,mm) over a flexible K-wire. Instruments were provided by Ulrich AG (approach) and Medacta International (spinal instrumentation). This

scenario introduces specular tools, transient occlusions, and view-dependent effects representative of clinical reality.

2.1 Acquisition rigs and imaging parameters

Camera settings For the SPP setup, 4 far-field and 8 near-field cameras (GoPro Hero 12 Black) were used. For the SPI setup, 4 far-field and 7 near-field cameras (GoPro Hero 12 Black) were used. All devices recorded at 4K resolution (9:16 aspect ratio) and 30 fps using the Linear lens mode. The white balance was fixed at 4500 K for all cameras. Far-field cameras operated with a shutter speed of 1/240 s and ISO 400. For near-field cameras, ISO was set to 400 for SPP and 100 for SPI, with a fixed shutter speed of 1/960 s.

Camera intrinsics Intrinsic parameters were estimated using a professional checkerboard [2]. Radial and tangential distortions were corrected, and undistorted images were used in all experiments.

Camera setup The camera locations are shown in Fig. 1. They were mounted mostly on walls, lamps and other operating room equipment as depicted in Fig. 2.

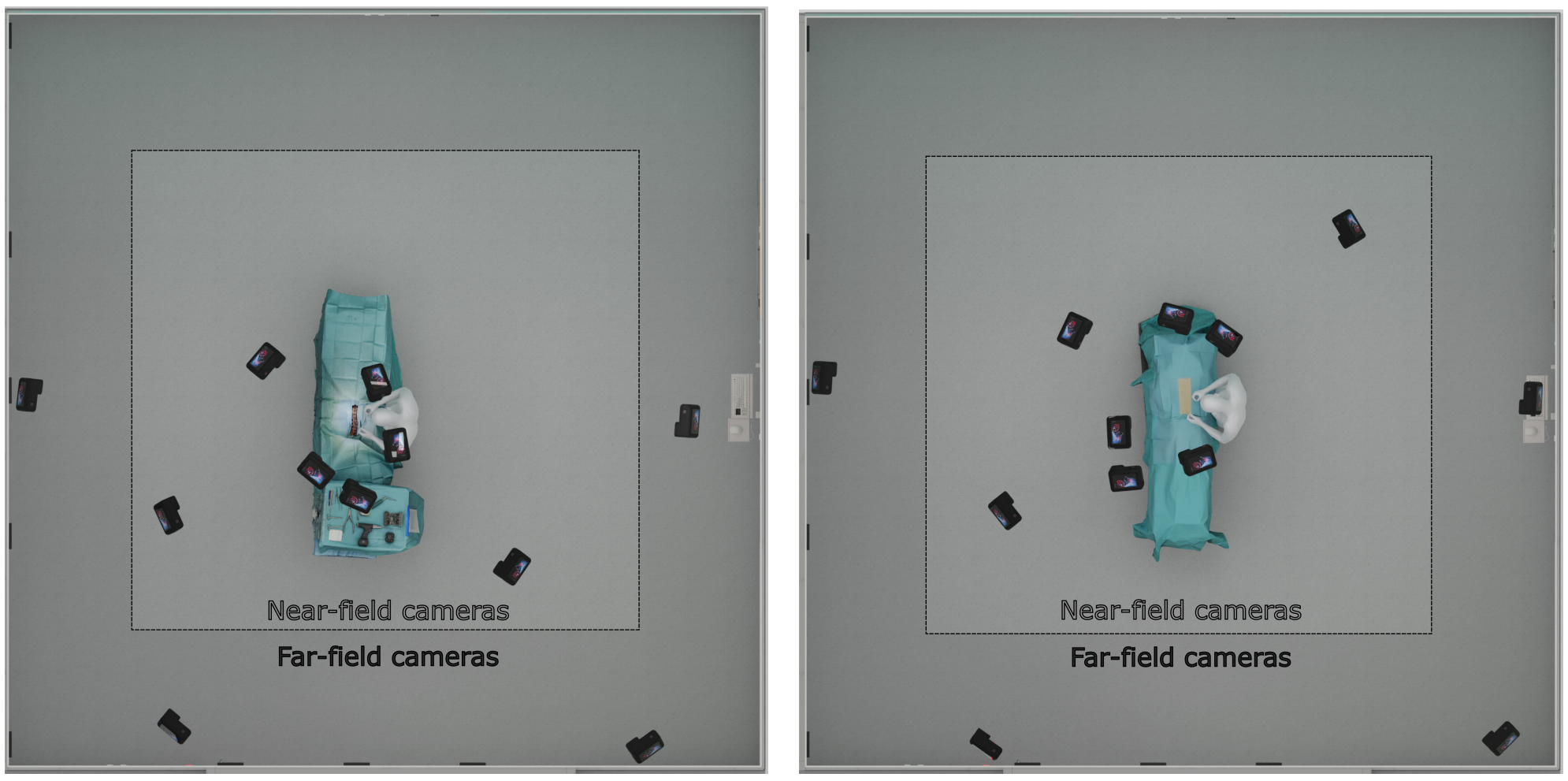

(a) (b)

Figure 1: Top-down view of camera locations for (a) SPP and (b) SPI. Cameras are visualized using a 4:1 GoPro HERO12 Black model for better visibility. Far-field cameras are mounted to the room walls, while near-field cameras are attached to lab equipment.

Camera extrinsics Both datasets provide synchronized video streams (temporal synchronization via [3]) and ground-truth camera poses from external calibration using

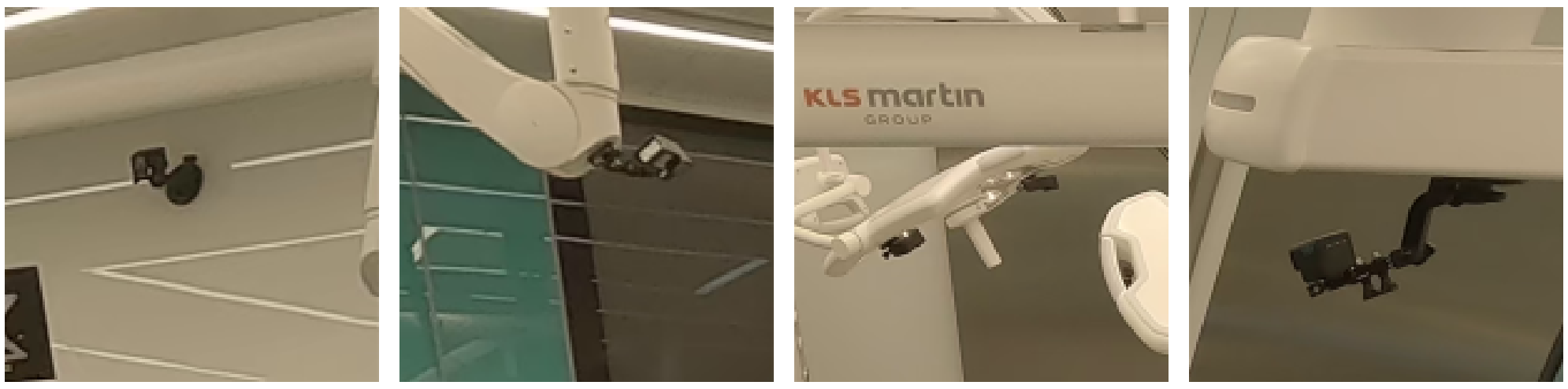

Figure 2: From left to right: far-field camera mounted on the wall, followed by three near-field cameras mounted on different sections of a surgical lamp within the operating room.

a moving checkerboard, a ChArUco board [4], and projected MSM [5], yielding mean reprojection errors of 0.46 pixel (SPP) and 0.40 pixel (SPI) over 2471 and 17811 mean per-camera correspondences, respectively.

2.2 Scene selection

We describe in the following section the selection of scenes used for analysis.

SPP1 Scene showing the surgeon performing the initial surgical approach. Standing on the right side of the patient, the surgeon uses a scalpel and surgical forceps to perform a superficial incision over the lumbar and thoracolumbar spine.

SPP2 Scene showing the surgeon organizing instruments and ensuring safety. The surgeon collects and folds gauze on the instrument table, then places a safety cover over the electrocautery tip before positioning the instrument back near the patient.

SPP3 Scene showing the surgeon preparing for the surgical approach. The surgeon retrieves a scalpel and forceps from the instrument table and turns toward the patient's right side to perform a superficial incision over the lumbar and thoracolumbar spine.

SPP4 Scene showing the surgeon positioning before the surgical approach. The surgeon moves along the patient's right side, turns toward the operative field, and palpates the sterile area over the lumbar spine to confirm the surgical site.

SPP5 Scene showing the surgeon initiating the skin incision. Standing on the patient's right side, the surgeon uses a scalpel and surgical forceps to perform the first superficial incision over the lumbar and thoracolumbar spine.

SPP6 Scene showing the surgeon completing the initial incision. Standing on the patient's right side, the surgeon finalizes the first skin incision and performs additional small cuts at the incision site using a scalpel and surgical forceps over the lumbar and thoracolumbar spine.

SPP7 Scene showing the surgeon performing hemostasis. Standing on the patient's

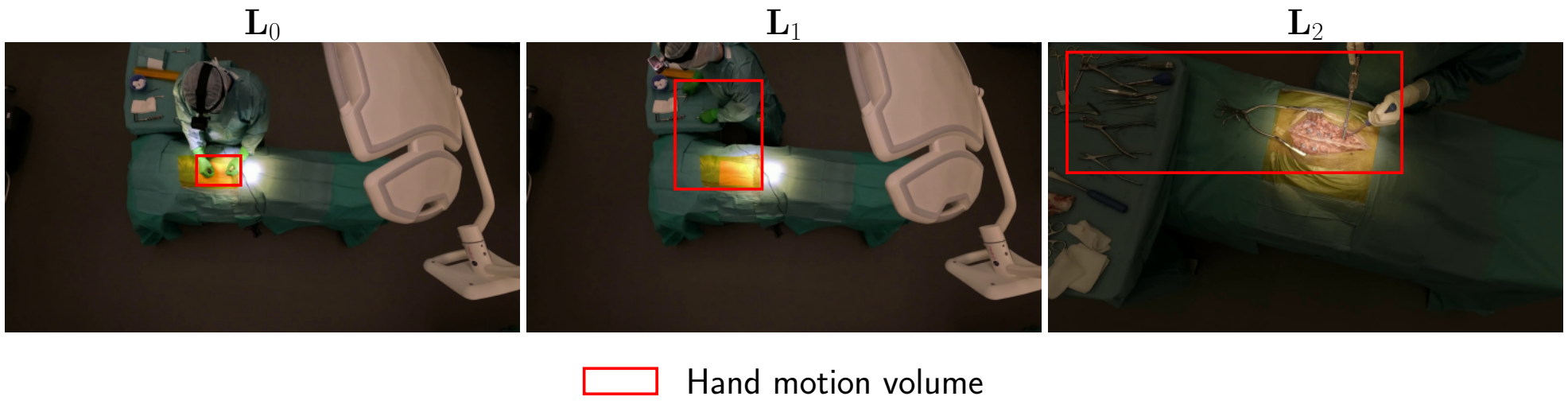

Figure 3: Visual comparison of complexity levels $\mathbf{L}_0$–$\mathbf{L}_2$. In $\mathbf{L}_0$, the surgeon utilizes small instruments with minimal hand motion. $\mathbf{L}_1$ introduces body motion, resulting in higher hand velocities. In $\mathbf{L}_2$ complexity is further increased through the use of larger tools and an expanded spatial range of hand motion.

right side, the surgeon uses surgical forceps to grasp bleeding vessels and applies a monopolar electrocautery instrument to the forceps tip to achieve coagulation.

SPI1 Scene showing the surgeon alternating between canal palpation and screw insertion preparation. The surgeon probes the pedicle canal for cortical breaches, retrieves the pedicle screwdriver with a mounted screw, verifies canal integrity once more, and positions the screwdriver at the predefined pedicle trajectory for screw placement.

SPI2 Scene showing the surgeon initiating pedicle screw insertion. The surgeon aligns the screwdriver with the pre-drilled pedicle trajectory while maintaining exposure with a Cobb elevator, briefly adjusts the elevator position, and then advances the pedicle screw into the vertebra.

The level of scene complexity comprises several characteristics. First, complexity varies with the surgical tools employed and the degree of occlusions occurring throughout the sequence. Second, the extent and dynamics of the surgeons' hand motion further contribute to overall complexity. This effect is visualized in Fig. 3, where larger motion volumes correspond to higher scene complexity, as operating over a broader workspace typically involves faster and less constrained movements. The scenes shown in Fig. 3 are taken from sequences SPP1, SPP3, and SPI1, representing complexity levels $\mathbf{L}_0$–$\mathbf{L}_2$, respectively. For a clearer visualization of these differences, we refer the reader to the Online Resource 2 video.

2.3 Ethics

SPP involved a non-invasive mock patient; no protected health information is present. SPI was conducted under ethics approval (BASEC Nr. 2021-01196).

2.4 Ground truth

The ground truth was generated using a custom Python-based annotation tool with a graphical user interface. This software allowed synchronized viewing of multi-camera video streams, enabling annotators to select specific frames and manually label the left and right hands independently.

To compute the 3D ground truth, we jointly optimize the 3D joint locations obtained by triangulation of the manually annotated 2D hand keypoints across multiple camera views, minimizing both the reprojection errors and an additional regularization that penalizes deviations between the predicted joint-to-joint distances and their estimated nominal bone lengths. For each timestamp t, let M_t denote the number of visible hands, and let $\mathcal{P}$ be the set of bone pairs (j,k) defining the hand kinematic structure. For each hand m and bone (j,k), the term minimizes the squared difference between the predicted bone length $\|\hat{\mathbf{X}}^h_{t,j} - \hat{\mathbf{X}}^h_{t,k}\|_2$ and the estimated reference length $\hat{l}^m_{j,k}$. The total loss is averaged over all bones, hands, and timestamps belonging to sequences involving the same surgeon:

$$L_{\text{bone}} = \frac{1}{\sum_{t=1}^{T} M_t \, |\mathcal{P}|} \sum_{t=1}^{T} \sum_{m=1}^{M_t} \sum_{(j,k)\in\mathcal{B}} \left(\left\| \hat{\mathbf{X}}^m_{t,j} - \hat{\mathbf{X}}^m_{t,k} \right\|_2 - \hat{l}^m_{j,k} \right)^2. \tag{7}$$

This term enforces consistent bone lengths across all timestamps and sequences recorded from the same surgeon, improving anatomical plausibility. Optimization is performed using the L-BFGS algorithm with strong Wolfe line search. The hyperparameters used were respectively set to $\lambda_{\text{reproj}} = 1$ and $\lambda_{\text{bone}} = 100$.

Inter-annotator variability To quantify the accuracy of our ground truth poses, we evaluated inter-annotator variability across 2D and 3D hand pose annotations. Four annotators independently labeled a total of 558 hands corresponding to six randomly selected timestamps and seen by 8 cameras among five (SPP1 – SPP5) sequences. For each annotated frame, pairwise Euclidean distances were computed between all annotator pairs for each corresponding joint, yielding both 2D pixel distances (after reprojection to camera space) and 3D distances (in millimeters) in the global coordinate system. These distances were then averaged across all frames and cameras to obtain an overall measure of variability per joint.

The results are shown in Fig. 4. Except for joints 1 and 2 (wrist and base of the palm), the inter-annotator variability remains below 6 pixels in 2D and below 6 mm in 3D, indicating high annotation consistency and labeling reliability. Joints 1 and 2, highlighted in grey in the histograms, were discarded from the ground truth due to their higher variability, which reflects inconsistent labeling unsuitable for quantitative evaluation.

Both the annotation software and the dataset will be released as open-source resources.

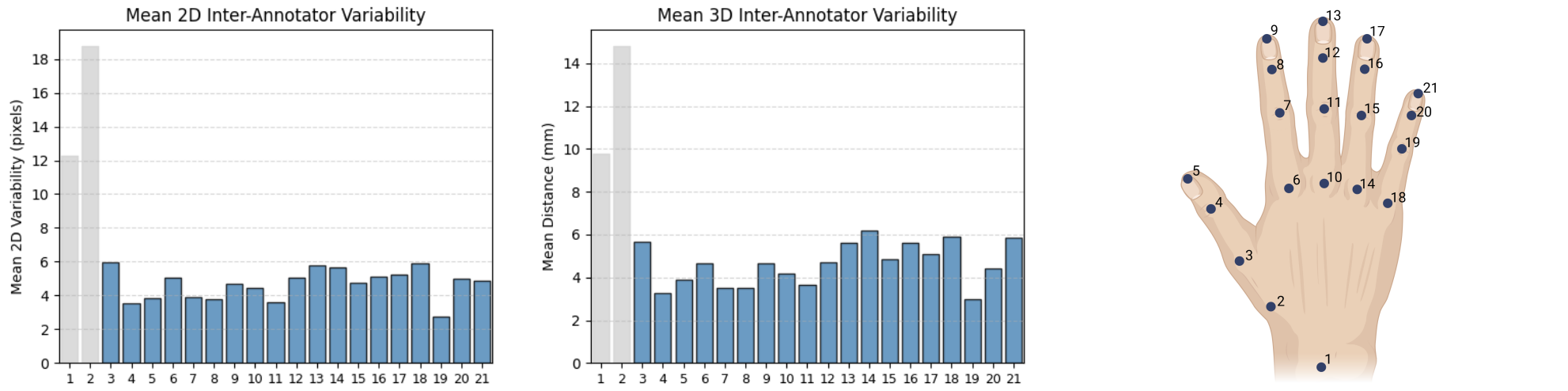

(a) 2D inter-annotator variability per joint (in pixels).

(b) 3D inter-annotator variability per joint (in mm).

(c) Hand joint model with 21 annotated keypoints. Created in BioRender.

Figure 4: **Inter-annotator variability across 2D and 3D annotations.** (a) 2D pixel-space variability aggregated across all eight cameras. (b) Mean 3D pairwise distances between all annotator pairs, computed per joint and averaged across all sequences and frames. (c) Illustration of the 21-joint hand model used for labeling and distance computation.

3 Additional results

This section presents additional quantitative and qualitative results that further demonstrate the performance and limitations of the proposed method.

Table 1 presents per-sequence quantitative results of the full pipeline. Each column of the table corresponds to a specific sequence among SPP or SPI and complexity level ($\mathbf{L}_0 - \mathbf{L}_2$). The reported metrics include the mean per-joint position error (MPJPE, in millimeters), mean reprojection error (MRE, in pixels), and the mean percentage of correct keypoints in 2D and 3D ($mPCK_{2D}$, $mPCK_{3D}$). Lower values of MPJPE and MRE indicate better spatial and projection accuracy, while higher $mPCK$ values reflect improved pose estimation consistency.

To highlight the improvements achieved by our full pipeline, Fig. 5 presents a qualitative comparison of 3D hand pose estimations from baseline methods and our approach.

To illustrate some limitations of our approach, Fig. 6 presents four qualitative examples of failure cases observed under challenging conditions (see caption for details).

3.1 Comparison with state-of-the-art monocular method

To contextualize our results with respect to 3D hand pose estimation, we evaluated the monocular HaMeR method [6] using the camera view with the least hand occlusion. HaMeR outputs 2D keypoints and a corresponding 3D MANO hand model [7], which encodes a statistical adult-sized hand prior. We conducted two experiments to evaluate the performance of this approach. First, we evaluated HaMeR in its default configuration without any modifications. Second, to address the depth and scale ambiguity inherent in monocular inference, we performed an experiment using knowledge

Data Collection Complexity	SPP 1 L_0	SPP 2 L_1	SPP 3 L_1	SPP 4 L_1	SPP 5 L_0	SPP 6 L_0	SPP 7 L_0	SPI 1 L_2	SPI 2 L_2	Mean
**MPJPE (mm)** ↓	8.3	9.1	10.2	8.1	8.8	8.5	7.0	7.6	8.8	8.5
**MRE (px)** ↓	6.0	8.2	9.0	8.7	7.3	7.2	6.9	9.8	10.3	8.2
$mPCK_{2D}$ ↑	83.6	74.6	74.9	76.0	77.9	78.8	79.9	72.9	70.3	76.6
$mPCK_{3D}$ ↑	76.1	70.7	71.8	77.2	71.7	74.0	79.8	77.5	73.4	74.7

Table 1: Full pipeline results on the surgical dataset showing MPJPE (mm), reprojection errrors (px), and mean PCK (%) of differing scenarions.

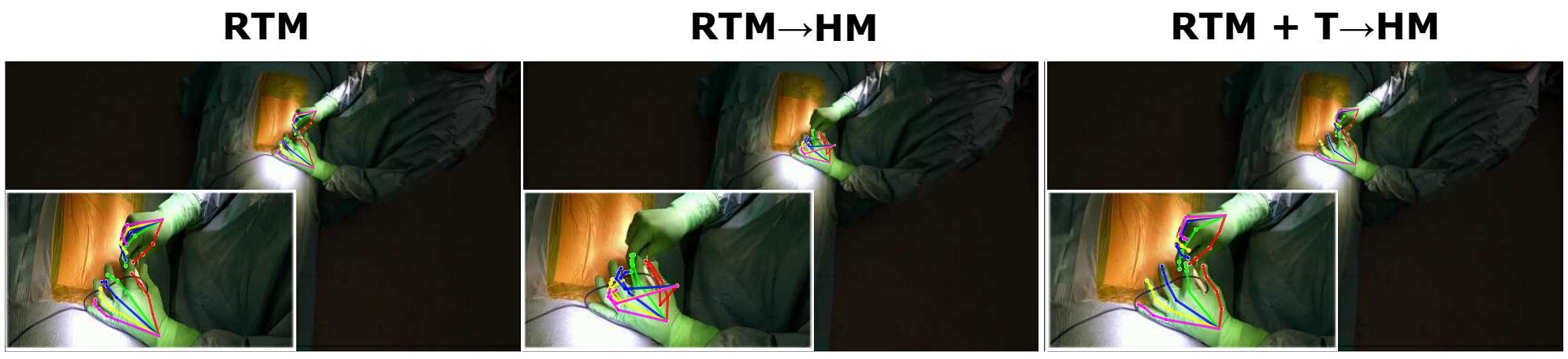

Figure 5: Qualitative comparison of 3D hand poses estimated using baseline methods and our approach (shown reprojected onto input images). From left to right: RTM Whole-Body, RTM → HM, and our method combining intermittent RTM, hand tracking, and HM. The first detects both hands but with coarse joints, the second misses one hand and mislocalizes joints, while ours accurately detects and localizes both hands.

Data Collection Complexity	SPP 1 L_0	SPP 2 L_1	SPP 3 L_1	SPP 4 L_1	SPP 5 L_0	SPP 6 L_0	SPP 7 L_0	SPI 1 L_2	SPI 2 L_2	Mean
**HaMeR (w/o scaling) [mm]** ↓	89.8	57.4	69.6	44.14	91.6	91.4	73.6	140.6	189.2	94.1
**HaMeR (w/ scaling) [mm]** ↓	51.5	49	46.9	72.1	44.8	47.0	44.9	44.4	44.7	49.5
**Ours [mm]** ↓	8.3	9.1	10.2	8.1	8.8	8.5	7.0	7.6	8.8	8.5

Table 2: Comparison of 3D hand pose estimation accuracy (MPJPE in mm). Results show that our method significantly outperforms the monocular HaMeR approach [6], even when the exact hand size is provided as input.

of the surgeon's actual hand size, information available from our 3D ground truth, to rescale the predicted output to the true physical dimensions. The resulting world-space joint configurations for both variants were compared to our 3D ground truth poses, with MPJPE (mm) reported in Table 2, with an illustrative example in Fig. 7.

Table 2 shows a substantial performance gap between the monocular baseline and our multi-view pipeline. Even with subject-specific scale correction, the monocular approach remains markedly less accurate, reflecting the inherent limitations of single-view inference. While rescaling improves global depth alignment, it cannot resolve perspective-dependent occlusions common in surgical scenes. In contrast, our method leverages multi-view triangulation, providing stronger geometric constraints and higher spatial accuracy, which is critical for high-precision clinical applications.

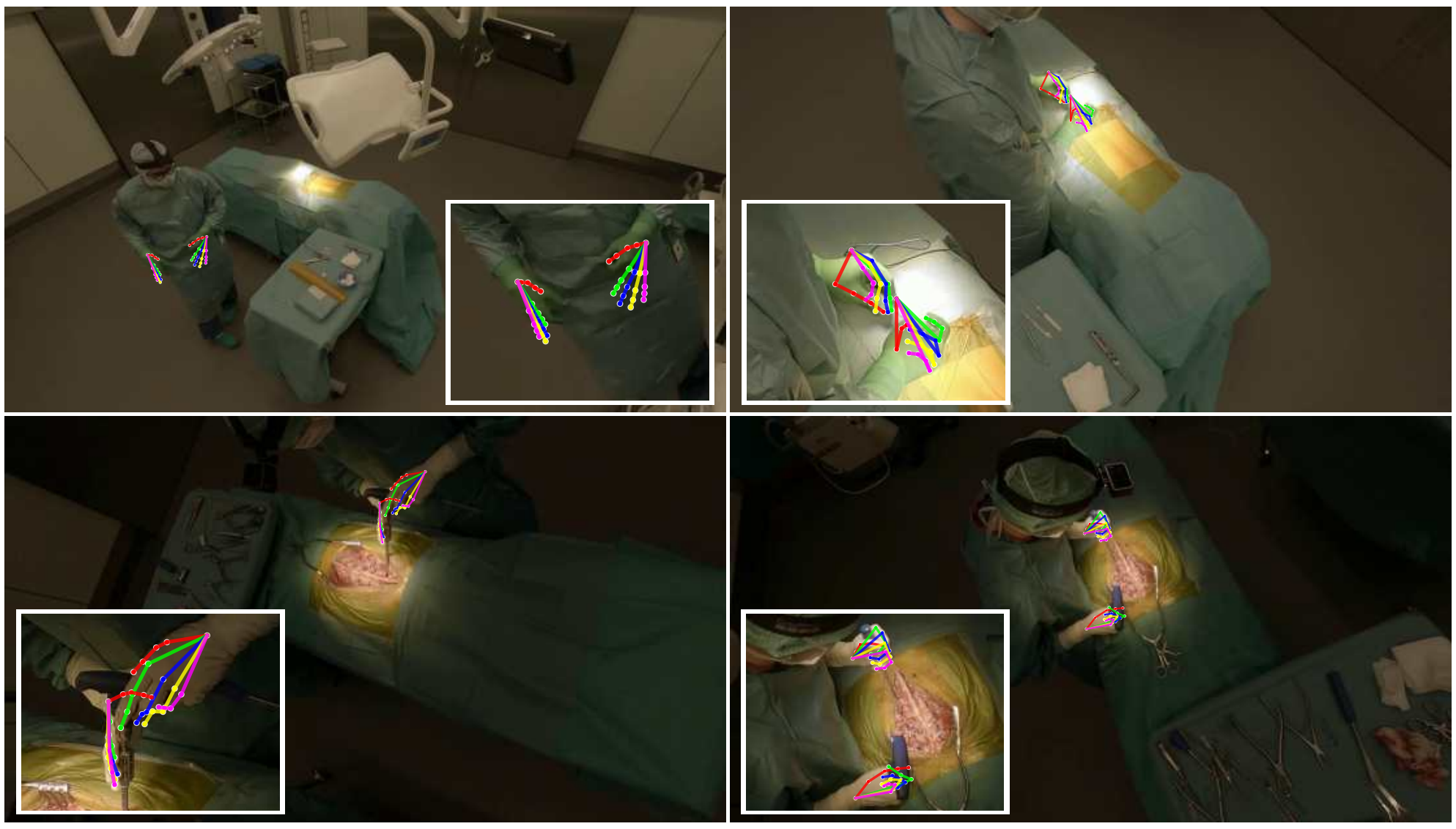

Figure 6: The main causes of failure include severe occlusions from surgical instruments or other objects, limited camera coverage (fewer than three active views), and strongly self-occluding hand poses such as closed fists.

4 Implementation details

All components of the proposed pipeline are built from publicly available pretrained models and run without any fine-tuning. Below, we summarize the key implementation details and hyperparameters for reproducibility.

2D detection and initial estimates. Person detection is performed using YOLOv11x [8]. As mentioned in the main paper, we assume a single-person scenario; therefore, for each frame, only the highest-confidence bounding box is retained for further processing. The RTMPose-Wholebody pose estimation model is then applied to these detections. The RTMPose-X [9] model weights and configuration was used for our implementation.

2D hand tracking and 2D hand pose estimation. For hand tracking, we employed the Efficient TAM-1B parameter model [10] in detecting hand mask and tracking throughout the scene. We take pretrained model weights and configuration files from the original paper. In terms of the 2D hand pose estimation model, we employ the 2D hand pose estimation RTMPose-M [9] model weights and configuration files.

Wrist confidence score For wrist keypoints, the confidence scores are set to 1.0, as empirical observations show that wrist detections are highly stable across frames. In the 3D optimization (Eq. 1 in the main paper), these confidence values weight the

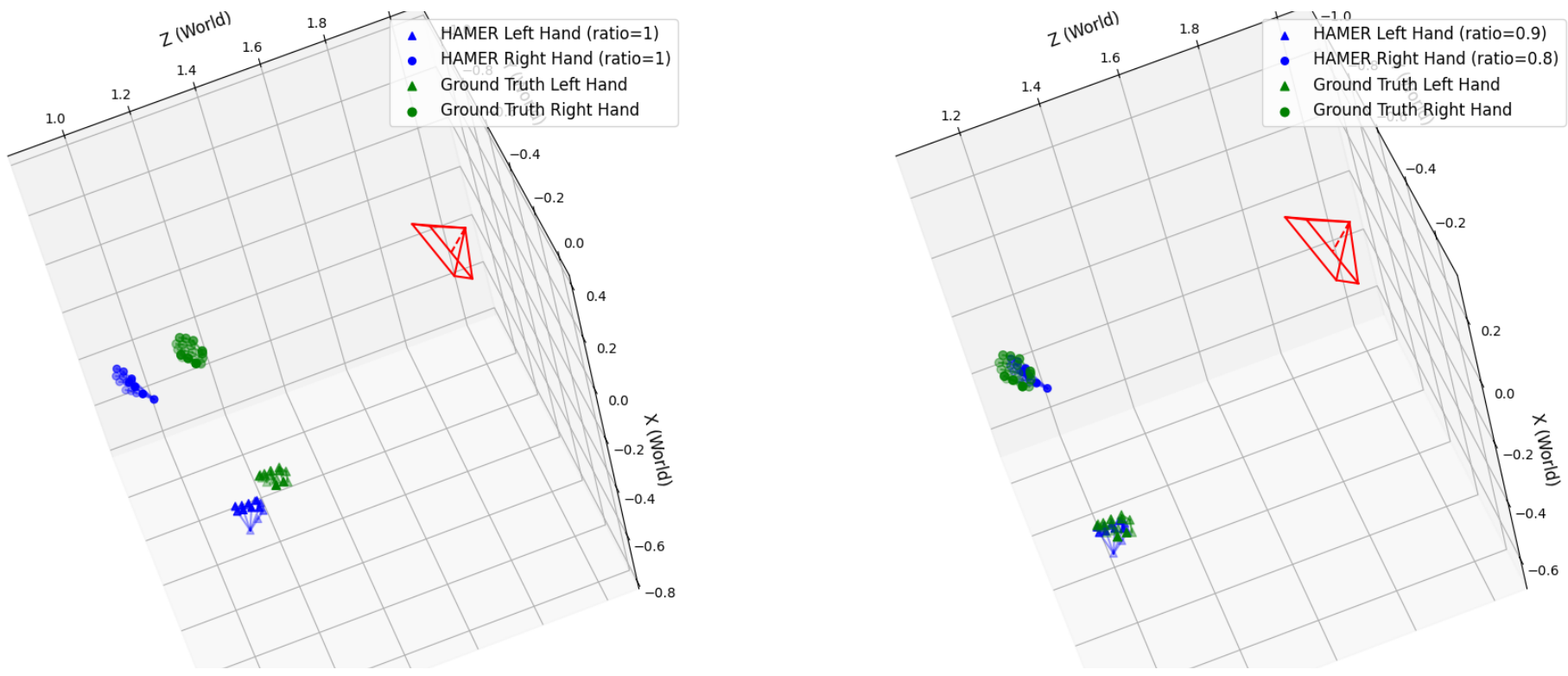

(a) Baseline HaMeR (No Scaling) (b) Scale-Corrected HaMeR

Figure 7: Comparison of 3D hand pose estimation using HaMeR [6]. (a) HaMeR estimation using the default MANO prior [7] (MPJPE: 87.2 mm). (b) HaMeR result after applying the subject-specific physical hand scale prior (MPJPE: 42.5 mm). Despite the scale adjustment in (b), our method achieves significantly higher accuracy with an MPJPE of 8.3 mm.

reprojection loss term; assigning a fixed high confidence effectively anchors the hand to the wrist position. This prevents temporal smoothness regularization from dominating during fast hand motions, ensuring accurate and stable wrist reconstruction.

3D pose estimation We employ the L-BFGS-B [11] optimizer (PyTorch v2.5.1) with a learning rate of 1.0, a maximum of 100 iterations, and a convergence tolerance of 1×10^{-5}. Optimization is performed on 50-frame windows with a 25-frame overlap to ensure temporal consistency. In our ablation study, the weighting factors are set to $\lambda_{\text{repr}} = 1.0$, $\lambda_{\text{smooth}} = 20.0$, $\lambda_{\text{shape}} = 50.0$, and $\lambda_{\text{biomech}} = 5.0$. For the final configuration, we exclude the biomechanical term ($\lambda_{\text{biomech}} = 0$), as it was found to significantly decrease 3D pose accuracy.

References

[1] Lin, T.-Y., Maire, M., Belongie, S., Bourdev, L., Girshick, R., Hays, J., Perona, P., Ramanan, D., Zitnick, C.L., Dollár, P.: Microsoft COCO: Common objects in context. In: European Conference on Computer Vision (ECCV) (2014). https://doi.org/10.1007/978-3-319-10602-1_48

[2] Professional checkerboard. https://calib.io/

[3] Meyer, J., Giraud, F., Wüthrich, J., Fürnstahl, P., Calvet, L.: Rocsync: Millisecond-accurate temporal synchronization for heterogeneous camera systems. arXiv preprint (2025)

[4] Garrido-Jurado, S., Muñoz-Salinas, R., Madrid-Cuevas, F.J., Marín-Jiménez, M.J.: Automatic generation and detection of highly reliable fiducial markers under occlusion. Pattern Recognition **47**(6), 2280–2292 (2014)

[5] Flückiger, T., Hein, J., Fischer, V., Fürnstahl, P., Calvet, L.: Automatic calibration of a multi-camera system with limited overlapping fields of view for 3d surgical scene reconstruction. International Journal of Computer Assisted Radiology and Surgery (2025)

[6] Pavlakos, G., Shan, D., Radosavovic, I., Kanazawa, A., Fouhey, D., Malik, J.: Reconstructing hands in 3D with transformers. In: CVPR (2024)

[7] Romero, J., Tzionas, D., Black, M.J.: Embodied hands: Modeling and capturing hands and their interactions. ACM Transactions on Graphics (ToG) **36**(6), 245–124517 (2017)

[8] Jocher, G., Qiu, J.: Ultralytics YOLO11

[9] Jiang, T., Lu, P., Zhang, L., Ma, N., Han, R., Lyu, C., Li, Y., Chen, K.: Rtmpose: Real-time multi-person pose estimation based on mmpose. arXiv preprint (2023)

[10] Xiong, Y., Zhou, C., Xiang, X., Wu, L., Zhu, C., Liu, Z., Suri, S., Varadarajan, B., Akula, R., Iandola, F., Krishnamoorthi, R., Soran, B., Chandra, V.: Efficient track anything. In: Proceedings of the IEEE/CVF International Conference on Computer Vision (ICCV) (2025)

[11] Zhu, C., Byrd, R.H., Lu, P., Nocedal, J.: Algorithm 778: L-bfgs-b: Fortran subroutines for large-scale bound-constrained optimization. ACM Trans. Math. Softw. **23**(4), 550–560 (1997) https://doi.org/10.1145/279232.279236
